# Unravelling the pathogenic role and genotype-phenotype correlation of the *USH2A* p.(Cys759Phe) variant among Spanish families

**DOI:** 10.1371/journal.pone.0199048

**Published:** 2018-06-18

**Authors:** Raquel Pérez-Carro, Fiona Blanco-Kelly, Lilián Galbis-Martínez, Gema García-García, Elena Aller, Blanca García-Sandoval, Pablo Mínguez, Marta Corton, Ignacio Mahíllo-Fernández, Inmaculada Martín-Mérida, Almudena Avila-Fernández, José M. Millán, Carmen Ayuso

**Affiliations:** 1 Department of Genetics, Instituto de Investigación Sanitaria–Fundación Jimenez Diaz University Hospital-Universidad Autónoma de Madrid (IIS-FJD, UAM), Madrid, Spain; 2 Center for Biomedical Network Research on Rare Diseases (CIBERER), ISCIII, Madrid, Spain; 3 Research group on Molecular, Cellular and Genomic Biomedicine, Health Research Institute La Fe (IIS La Fe), Valencia, Spain; 4 Department of Ophthalmology, Instituto de Investigación Sanitaria–Fundación Jimenez Diaz University Hospital–Universidad Autónoma de Madrid (IIS-FJD, UAM), Madrid, Spain; 5 Department of Epidemiology and Biostatistics, Instituto de Investigación Sanitaria-Fundación Jimenez Diaz-Universidad Autónoma de Madrid (IIS-FJD, UAM), Madrid, Spain; Hebrew University Hadassah Medical School, ISRAEL

## Abstract

**Introduction:**

Mutations in *USH2A* cause both isolated Retinitis Pigmentosa (RP) and Usher syndrome (that implies RP and hearing impairment). One of the most frequent variants identified in this gene and among these patients is the p.(Cys759Phe) change. However, the pathogenic role of this allele has been questioned since it was found in homozygosity in two healthy siblings of a Spanish family. To assess the causative role of *USH2A* p.(Cys759Phe) in autosomal recessive RP (ARRP) and Usher syndrome type II (USH2) and to establish possible genotype-phenotype correlations associated with p.(Cys759Phe), we performed a comprehensive genetic and clinical study in patients suffering from any of the two above-mentioned diseases and carrying at least one p.(Cys759Phe) allele.

**Materials and methods:**

Diagnosis was set according to previously reported protocols. Genetic analyses were performed by using classical molecular and Next-Generation Sequencing approaches. Probands of 57 unrelated families were molecularly studied and 63 patients belonging to these families were phenotypically evaluated.

**Results:**

Molecular analysis characterized 100% of the cases, identifying: 11 homozygous patients for *USH2A* p.(Cys759Phe), 42 compound heterozygous patients (12 of them with another missense *USH2A* pathogenic variant and 30 with a truncating *USH2A* variant), and 4 patients carrying the p.(Cys759Phe) allele and a pathogenic variant in another RP gene (*PROM1*, *CNGB1* or *RP1*). No additional causative variants were identified in symptomatic homozygous patients. Statistical analysis of clinical differences between zygosity states yielded differences (p≤0.05) in age at diagnosis of RP and hypoacusis, and progression of visual field loss. Homozygosity of p.(Cys759Phe) and compound heterozygosity with another *USH2A* missense variant is associated with ARRP or ARRP plus late onset hypoacusis (OR = 20.62, CI = 95%, p = 0.041).

**Conclusions:**

The present study supports the role of *USH2A* p.(Cys759Phe) in ARRP and USH2 pathogenesis, and demonstrates the clinical differences between different zygosity states. Phenotype-genotype correlations may guide the genetic characterization based upon specific clinical signs and may advise on the clinical management and prognosis based upon a specific genotype.

## Introduction

Retinitis Pigmentosa (RP; *MIM#268000*) is the most common form of Inherited Retinal Dystrophies (IRD),with a prevalence of approximately 1 in 4000 [1]. It is characterized by primary degeneration of the rods in the early stage of the disease, with progressive evolution and, currently, without a treatment, leading to visual impairment or blindness [2]. Night blindness is the first symptom, followed by constriction of the peripheral visual field, and slow and progressive decrease of central vision [3]. RP is highly heterogeneous, both clinically and genetically. RP can be a non-syndromic disease which represents 70–80% of RP cases or it can be associated with other systemic alterations (syndromic RP; 20–30%) [4]. Usher syndrome (USH), the most frequent IRD syndromic disorder, is defined by sensorineural hearing loss together with RP. The three clinical subtypes: Usher syndrome type I (USH1) (*MIM#276901*), type II (USH2) (*MIM#276902*) and type III (USH3) (*MIM#276903*) are distinguished depending on the severity and onset of visual impairment and hearing loss, and on the presence of vestibular impairment [5].

The *USH2A* gene encodes for a transmembrane protein with a large extracellular portion containing 10 laminin EGF-like domains, 35 fibronectine type-III motifs as well as two laminin G domains [6,7] and is expressed in adult human retina, specially localized to the photoreceptor cells and in fetal human cochlea and eye [8–10]. Pathogenic variants in *USH2A* have been associated with both non-syndromic autosomal recessive RP (ARRP, 10–15% of the characterized cases) and USH2 (80%) [11–13], being the p.(Cys759Phe) one of the most frequent pathogenic variants in the Spanish population. This variant accounts for 4.5% of the RP cases [12,14] and for 8.1% of the USH2 patients [15]. In most cases it has been observed as an autosomal recessive inherited condition, although a rare case of uniparental isodisomy has been described [16], demonstrating that other mechanisms are possible.

The high prevalence of the p.(Cys759Phe) variant prompted us to determine the implication of this variant and to elucidate whether it is the cause of syndromic and non-syndromic RP in our cohort or otherwise it is a random association or a modifier variant of RP and USH2 [17].

## Results

### Spanish cohort carrying p.(Cys759Phe) pathogenic variant

A total of 57 probands belonging to Spanish families affected with non-syndromic ARRP or USH2, carrying the p.(Cys759Phe) variant either in homozygosis (11/57) or heterozygosis (46/57), were selected. These last included both patients in which only one p.(Cys759Phe) allele was found (17) and 29 patients previously characterized with a second allele using classical molecular techniques (ARRP/Usher genotyping microarray, Sanger sequencing or MLPA analysis).

In order to find the second mutated allele or to discard the implication of other causative genes in the pathology, we analyzed, by different targeted Next-Generation Sequencing (NGS) approaches, not only the non-fully characterized heterozygous families (17/57) but also the probands of the p.(Cys759Phe) homozygous families (11/57) and 24 of the 29 compound heterozygous probands previously characterized by classical molecular techniques. The non-analyzed heterozygotes by NGS were 3 compound heterozygous patients with no further sample available (RP-1574, RP-0391 and RP-1016/982) and 2 carriers of p.(Cys759Phe) allele characterized with other RD genes by classical molecular tests (RP-1899 and RP-0551).

After both classical and NGS studies, we could characterize 53 families with two mutations in the *USH2A* gene, including 11 homozygous and 42 compound heterozygous. Whenever co-segregation studies were possible (46 out of 53 families) *USH2A* variants co-segregated with the disease (pedigrees of homozygous and compound heterozygous families are shown in S1A and S1B Fig, respectively). A second *USH2A* mutated allele was found by NGS in 15 heterozygous cases, being 3 of them novel variants (Table 1). No other causative gene among the homozygous cases was found. In 4 additional cases, out of 46 heterozygous cases, pathogenic variants in *RP1*(2), *PROM1* and *CNGB1* genes were identified as cause of the disease, co-segregating in the families with RP (S1C Fig). Therefore, these patients are only carriers for *USH2A* p.(Cys759Phe) (Table 1). Among the compound heterozygous patients initially characterized by classical molecular techniques and re-analyzed using NGS, we found additional variants in the following families:

i) In family RP-0004 an unreported likely pathogenic variant in *PCDH15* was found: c.124G>T; p.(Gly42*). No second allele was detected by NGS analysis (Clinical Exome Solution; Sophia Genetics), which also includes copy number variations (CNV) detection.
ii) RP-0810 also carried the *USH2A* variant c.12574C>T; p.(Arg4192Cys).This variant has been previously reported as pathogenic in a family with ARRP [18]. Segregation analysis in family RP-0810 showed that the variant was *in cis* with the p.(Cys759Phe) allele (S1B Fig). Both missense *USH2A* variants -p.(Cys759Phe) and p.(Arg4192Cys)- were *in trans* with a third *USH2A* truncating variant p.(Thr2812Metfs*17).
iii) RP-0605 was a compound heterozygous family for *USH2A* variants p.(Cys759Phe) and p.(Lys811Aspfs*11). Using NGS, we additionally detected the *CNGB3* c.852+1G>C variant in homozygous state. This variant was previously reported by Mayer *et al*. [19] in two families suffering from achromatopsia. Re-evaluation of the clinical history of our patient suggested the presence of two concurrent retinal pathologies. On the one hand, proband of the family RP-0605 is affected by RP+ hypoacusis. On the other hand, patient suffered from congenital nystagmus and reported photophobia and low VA since 3 years old. These latter symptoms and signs may be in keeping with a concurrent diagnosis of a cone dystrophy or an achromatopsia.

**Table 1:** Genotype of 46 Spanish families carrying the p.(Cys759Phe) pathogenic variant in compound heterozygous state or heterozygous carriers with other causative genes.

**Category**^a^	**Family ID**	**Patient ID**	**Technique (A2)**	**Gene**	**Nucleotide change (A2)**	**Amino acid change (A2)**	**Zygosis**	**Reference**
**Category B** (Cys759Phe+*USH2A* missense)	RP-0366	96/0881	Targeted-NGS	*USH2A*	c.754G>T	p.(Gly252Cys)	Heterozygous	[20]
	RP-1979	12/1337	Targeted-NGS	*USH2A*	c.1606T>C	p.(Cys536Arg)	Heterozygous	[21]
	**RP-1053**	**06/0127**	**Targeted-NGS**	*USH2A*	**c.3507G>C**	**p.(Trp1169Cys)**	Heterozygous	**This study**
	RP-0721	02/0555	Sanger	*USH2A*	c.3713C>G	p.(Thr1238Arg)	Heterozygous	[11]
	RP-2113	13/0464	Targeted-NGS	*USH2A*	c.5462A>G	p.(Lys1821Arg)	Heterozygous	[22]
	**RP-2504**	**15/2443**	**Clinical exome**	*USH2A*	**c.9389G>T**	**p.(Trp3130Leu)**	Heterozygous	**This study**
	RP-0752	02/1128	Usher microarray	*USH2A*	c.9799T>C	p.(Cys3267Arg)	Heterozygous	[23]
	RP-2156	13/1196	ARRP microarray	*USH2A*	c.9799T>C	p.(Cys3267Arg)	Heterozygous	[23]
	RP-2494	15/2242	Clinical exome	*USH2A*	c.9799T>C	p.(Cys3267Arg)	Heterozygous	[23]
	RP-2372	14/1933	ARRP microarray	*USH2A*	c.11156G>A	p.(Arg3719His)	Heterozygous	[24]
	RP-0653	01/0385	Sanger	*USH2A*	c.12575G>A	p.(Arg4192His)	Heterozygous	[11]
	RP-1574	10/0653	ARRP microarray	*USH2A*	c.13010C>T	p.(Thr4337Met)	Heterozygous	[23]
**Category C** (Cys759Phe+*USH2A* truncating)	RP-1525	09/2102	ARRP microarray	*USH2A*	c.100C>T	p.(Arg34*)	Heterozygous	[21]
	RP-0391	97/0318	ARRP microarray	*USH2A*	c.187C>T	p.(Arg63*)	Heterozygous	[21]
	RP-1802	11/1105	Targeted-NGS	*USH2A*	c.920_923dupGCAA	p.(His308Glnfs*16)	Heterozygous	[6]
	**RP-0016**	**934**	**Sanger**	*USH2A*	**c.944_951dupCACAGCGG**	p. (Cys318Hisfs*21)	Heterozygous	[25]
	RP-1412	09/0426	Targeted-NGS	*USH2A*	c.1214delA	p.(Asn405Ilefs*3)	Heterozygous	[26]
	RP-0004	0729	**Sanger**	*USH2A*	c.2135delC	p.(Ser712*)	Heterozygous	[25]
	RP-0879	04/0740	ARRP microarray	*USH2A*	c.2135delC	p.(Ser712*)	Heterozygous	[25]
	RP-1104	06/0998	ARRP microarray	*USH2A*	c.2299delG	p.(Glu767Serfs*21)	Heterozygous	[8]
**Category**^a^	**Family ID**	**Patient ID**	**Technique (A2)**	**Gene**	**Nucleotide change (A2)**	**Amino acid change (A2)**	**Zygosis**	**Reference**
**Category C** (Cys759Phe+*USH2A* truncating)	RP-1590	10/0779	ARRP microarray	*USH2A*	c.2299delG	p.(Glu767Serfs*21)	Heterozygous	[8]
	RP-1810	11/1176	ARRP microarray	*USH2A*	c.2299delG	p.(Glu767Serfs*21)	Heterozygous	[8]
	RP-1858	11/1929	ARRP microarray	*USH2A*	c.2299delG	p.(Glu767Serfs*21)	Heterozygous	[8]
	RP-2130	13/0792	ARRP microarray	*USH2A*	c.2299delG	p.(Glu767Serfs*21)	Heterozygous	[8]
	RP-0605^b^	00/0554	ARRP microarray	*USH2A*	c.2431_2432delAA	p.(Lys811Aspfs*11)	Heterozygous	[27]
	RP-0610	00/0505	ARRP microarray	*USH2A*	c.2431_2432delAA	p.(Lys811Aspfs*11)	Heterozygous	[27]
	RP-1817	11/1316	ARRP microarray	*USH2A*	c.2431_2432delAA	p.(Lys811Aspfs*11)	Heterozygous	[27]
	**RP-1016/RP-982**	**05/1231**	**MLPA**	*USH2A*	**del Ex.22-29**	** **	Heterozygous	**This study**
	RP-0467	05/0084	Sanger	*USH2A*	c.7595-2144A>G	p.(Lys2532Thrfs*56)	Heterozygous	[28]
	RP-1031	05/1440	Sanger	*USH2A*	c.7595-2144A>G	p.(Lys2532Thrfs*56)	Heterozygous	[28]
	RP-1776	11/0774	Sanger	*USH2A*	c.7595-2144A>G	p.(Lys2532Thrfs*56)	Heterozygous	[28]
	RP-2262	14/0248	Targeted-NGS	*USH2A*	c.7595-2144A>G	p.(Lys2532Thrfs*56)	Heterozygous	[28]
	RP-0810^c^	03/0809	Sanger	*USH2A*	c.8435_8438delCCTA	p.(Thr2812Metfs*17)	Heterozygous	[23]
	RP-0385	10/0930	MLPA	*USH2A*	del Ex.44		Heterozygous	[29]
	RP-2424	15/0499	Clinical exome	*USH2A*	c.10759C>T	p.(Gln3587*)	Heterozygous	[30]
	RP-0061	05/0540	Targeted-NGS	*USH2A*	c.11548+2T>G	Splicing defect	Heterozygous	[30]
	RP-2089	13/0144	ARRP microarray	*USH2A*	c.11864G>A	p.(Trp3955*)	Heterozygous	[7]
	**RP-2529**	**15/1890**	**Clinical exome**	*USH2A*	**c.12457delG**	p.(Ala4153Profs*14)	Heterozygous	**This study**
	RP-1059	06/0896	Sanger	*USH2A*	c.13745delT	p.(Ile4582Lysfs*14)	Heterozygous	[11]
	RP-1422	09/0610	Clinical exome	*USH2A*	c.13811+2T>G	splicing defect	Heterozygous	[31]
	RP-2396	14/2336	Targeted-NGS	USH2A	c.14091delT	p.(Phe4697Leufs*2)	Heterozygous	[32]
	RP-0784	03/0735	Clinical exome	USH2A	c.14180G>A	p.(Trp4727*)	Heterozygous	[13]
**Category**^a^	**Family ID**	**Patient ID**	**Technique (A2)**	**Gene**	**Nucleotide change (A2)**	**Amino acid change (A2)**	**Zygosis**	**Reference**
**Other genes**	RP-1914	12/0131	Targeted-NGS	*CNGB1*	c.2957A>T	p.(Asn986Ile)	Homozygous	[33]
	RP-1899	11/2421	ARRP microarray	*PROM1*	c.1354dupT	p.(Tyr452Leufs*13)	Homozygous	[34]
	RP-0551	05/1342	Sanger	*RP1*	c.1625C>G	p.(Ser542*)	Homozygous	[35]
	RP-1772^d^	11/0727	Targeted-NGS	*RP1*	c.2431delA	p.(Ser812Valfs*36)	Heterozygous	[22]

^a^Patients were organized into different categories: compound heterozygous p.(Cys759Phe) + *USH2A* missense mutation (Category B), compound heterozygous p.(Cys759Phe) + *USH2A* truncating mutation (Category C), and "Other genes", carriers for p.(Cys759Phe) allele + causative mutation(s) in other RP gene.

^b^A splicing variant in *CNGB3* (c.852+1G>C) in homozygosity was detected in the proband after NGS-reanalysis.

^c^A third *USH2A* variant [c.12574C>T; p.(Arg4192Cys] was detected *in cis* with p.Cys759Phe, after NGS re-analysis.

^d^The RP-1772 family was re-classified as autosomal dominant RP.

Abbreviations: A2, second allele detected in *USH2A* or other RP gene; Technique (A2), technique by which the second mutation was detected; ARRP microarray, genotyping microarray for autosomal recessive retinitis pigmentosa.

Novel variants in *USH2A* are displayed in bold.

Variants found in both genes (*USH2A* and *CNGB3*) segregated in the family; therefore suggesting a RP diagnosis due to *USH2A*, and a likely cone dystrophy/achromatopsia diagnosis due to *CNGB3* in the proband; and a cone dystrophy/achromatopsia diagnosis in his brother (sibling II:2, S1B Fig).

### *In silico* predictions of mutated Cys759Phe Usherin

The Cys759 is a highly conserved position (98.16% in an alignment of 489 proteins, according to USMA tool) located inside a EGF laminin, a globular domain described to enhance USH2A stability in the basement membrane by prompting its interaction with collagen IV [36]. The substitution of the cysteine at position 759 by a phenylalanine would also disrupt a predicted disulfide bridge with Cys477 [12] as a source of erroneous protein folding and instability, as it is reported by USMA and Uniprot database.

In addition to the idea of the variant affecting a functional residue, the contiguous residue is glycosylated in a usherin orthologous protein (from fruit-fly) [37]. This has been used to predict a glycosylation site in USH2A Asn760 residue, based on residue conservation [38]. N-linked glycosylations are known to play a role in protein correct folding and cell-extracellular matrix attachment. The potential glycosylation at that position could be lost in the variant p.(Cys759Phe).

We also explored the functions that would be affected by the alteration of USH2A functionality. Thus, using the STRING database [39], we found 48 Gene Ontology terms (biological process) over-represented (False Discovery Rate, FDR<0.05), mainly related to ear and eye morphogenesis in the proteins interacting with USH2A (S1 Table).

### Genotype-phenotype correlation

In order to ease the comprehension of the p.(Cys759Phe) variant phenotype analysis, we divided those patients with the p.(Cys759Phe) allele into 3 genotype categories: i) Category A, all fourteen homozygous patients for the variant; ii) Category B, fourteen compound heterozygous patients with the p.(Cys759Phe) and a missense pathogenic variant; and iii) Category C, thirty-one patients with the p.(Cys759Phe) and, additionally, a truncating (nonsense, indels, deep-intronic and canonical splice site) variant. Patients presenting a causative variant in a different RP gene (not *USH2A*) were not included in these categories.

Based on the compiled clinical information, all the patients were assigned to one of the following three groups: ARRP or sporadic RP (ARRP/SRP), USH2 or RP + hypoacusis (when the available data were not sufficient for classification as USH2). Definition for this classification is detailed in "Clinical examination", in the "Material and methods" section. Ophthalmological data were available for 63 patients (56 families), including patients with mutations only in *USH2A* (59 cases from 52 families) and patients with mutations in other RP genes (4 cases from 4 families). Data on hearing loss were available for 52 patients (belonging to 45 families) with mutations only in *USH2A* and for 4 patients (4 families) with mutations in non-*USH2A* genes.

The number and percentage of patients classified according to the clinical subtype and genotype are shown in Table 2.

**Table 2:** Likelihood of presenting a specific clinical diagnosis for patients carrying p.(Cys759Phe) variant based on their genotype.

Diagnosis	Category A: p.(Cys759Phe) Homozygous		Category B: Compound Heterozygous p.(Cys759Phe) +*USH2A* missense		Category C: Compound Heterozygous p.(Cys759Phe) +*USH2A* truncating		Category A vs Category B	Category A vs Category C	Category B vs Category C
	N	%	N	%	N	%	likelihood of presenting Usher II diagnosis (p value, two-tailed Fisher´s test)		
**Usher syndrome type II**	0	0	0	0	8	25.8	NA	**0.043**	**0.043**
**ARRP/SRP**	9	64.3	9	64.3	19	61.3
**RP+hypoacusis**	5	35.7	5	35.7	4	12.9
**Total**	14	100	14	100	31	100

Fifty-nine cases belonging to 52 families were included in the analysis (4 families having causative mutations in other RP genes and presenting only the Cys759Phe allele in *USH2A* were excluded; also the proband of the family RP-2424, since only molecular information was available).

To facilitate the comprehension of the genotype-phenotype correlation analysis, the patients were classified into three different categories: homozygous patients for p.(Cys759Phe) variant (Category A); compound heterozygous patients carrying additionally other *USH2A* variant (missense, Category B and truncating, Category C).

The likelihood of presenting an USH2 diagnosis for each category was calculated by two-tailed Fisher´s test.

Abbreviations: RP, Retinitis Pigmentosa; AR, autosomal recessive; S, sporadic; NA, non applicable.

Complete clinical information of the studied patients is shown in S2 Table.

Patients presenting an *USH2A* truncating mutation in compound heterozygosity with p.(Cys759Phe) (Category C) were likely to present an USH2 diagnosis when compared with homozygous patients (Category A), or heterozygous patients from Category B (p = 0.043 in both cases, Table 2).

Additionally, there was more than a 20 fold increased chance (OR = 20.617 (1.130–376.212)) of presenting a non USH2 diagnosis when patients do not have a truncating variant in addition to p.(Cys759Phe) (p = 0.041, Table 3). The sensitivity and negative predictive value were both 100%.

**Table 3:** Likelihood of presenting a RP or RP + hypoacusis instead of a USH2 diagnosis in patients which carry a *USH2A* missense variant, comparing them with patients with an *USH2A* truncating variant, in addition to p.(Cys759Phe).

Diagnosis	Category A+B		Category C		Category A+B vs Category C
	N	%	N	%	likelihood of presenting a RP or RP+hypoacusis diagnosis (IC-95%)
**Usher syndrome type II**	0	0	8	25.8	
**ARRP/SRP + RP+hypoacusis**	28	100	23	74.2	OR = 20.617 (1.130–376.212)[40]; p = 0.041
**Total**	28	100	31	100

Fifty-nine cases belonging to 52 families were included in the analysis (4 families having causative mutations in other RP genes and presenting only the Cys759Phe allele in *USH2A* were excluded; also the proband of the family RP-2424, since only molecular information was available).

Abbreviations: IC, interval of confidence; RP, retinitis pigmentosa; AR, autosomal recessive; S, sporadic; O, odds ratio.

The mean and standard deviation (SD) of the analysed phenotype features for patients with RP, RP+hypoacusis or USH2, distributed by genotype categories, are shown in Table 4.

**Table 4:** Phenotypic findings in 59 patients from 52 families carrying the p.(Cys759Phe) variant, classified by their genotype.

	Age at diagnosis (yr)	NB onset (yr)	VF loss onset (yr)	VF, age at measurement (yr)	VF (degrees)	VA loss onset (yr)	VA, age at measurement (yr)	Best Eye Acuity (decimal)	Cataract, age at diagnosis (yr)	Hypoacusis age at diagnosis (yr)
**Category A: p.(Cys759Phe) Homozygous**	43.1±10.7 (N = 7)	29.5±10.0 (N = 11)	31.0±10.0 (N = 12)	49.9±19.9 (N = 11)	12.1±7.3 (N = 12)	41.5±11.0 (N = 6)	46.1±20.6 (N = 12)	1.0±1.3 (N = 12)	54.2±10.3 (N = 5)	72.5±3.5 (N = 6)
**Category B: Compound Heterozygous p.(Cys759Phe)+*USH2A* missense**	43.9±10.7 (N = 8)	25.8±13.5 (N = 13)	27.8±11.4 (N = 12)	42.9±8.9 (N = 13)	13.0±10.8 (N = 13)	37.8±13.3 (N = 6)	49.1±9.9 (N = 9)	0.5±3.4 (N = 10)	45.2±10.6 (N = 9)	31.3±20.1 (N = 4)
**Category C: Compound Heterozygous p.(Cys759Phe)+*USH2A* truncating**	29.5±12.0 (N = 20)	26.5±9.7 (N = 25)	28.0±8.2 (N = 21)	40.6±12.8 (N = 21)	12.5±7.7 (N = 20)	31.7±9.4 (N = 15)	43.5±13.0 (N = 20)	0.7±0.3 (N = 21)	49.0±12.1 (N = 10)	23.6±17.2 (N = 7)
**Category A vs Category B+C**	0.069	0.362	0.365	0.205	0.922	0.149	0.896	0.373	0.230	**<0.001**
**Category A vs Category C**	**0.016**	0.410	0.394	0.181	0.885	0.089	0.701	0.469	0.408	**<0.001**
**Category A vs Category B**	0.897	0.442	0.465	0.295	0.417	0.614	0.661	0.232	0.159	**0.023**
**Category B vs Category C**	**0.007**	0.861	0. 938	0.554	0.523	0.334	0.215	0.151	0.479	0.547
**Category A+B vs Category C**	**0.001**	0.755	0.639	0.194	0.628	0.069	0.408	0.787	0.907	0.126

Families with mutations in other RD genes and patient with neither ophthalmological nor audiological available data (RP-2424) were excluded. Mean and SD values are displayed for each genetic category and phenotypic trait (Student’s t test). The number of patients included in the statistical analysis is indicated in brackets.

Abbreviations: NB, night blindness; VF, visual field; VA, visual acuity. Statistical significant differences between different categories are marked in bold (p≤ 0.05).

The analysis revealed statistically significant differences between patients belonging to Category C compared with the other groups (Categories A and/or B), for age at diagnosis (p = 0.001, Category A+B vs Category C). Carrying a truncating mutation was associated to an earlier age of diagnosis and age of onset of VA loss, and hypoacusis diagnosis tended to be earlier, although these differences did not become statistically significant (p = 0.069 and p = 0.126, Category A+B vs Category C, respectively). For all categories, VA loss appeared late in the disease´s evolution, being ≥0.4 (decimal) until the fifth decade of life.

The main noticed difference within patients was in age at diagnosis of hypoacusis. Patients from categories B and C are significantly more likely to present an earlier diagnosis of hearing loss than patients from category A (p = 0.023 and p<0.01, respectively), being the truncating group the one with an earlier diagnosis. Five of the patients presenting the p.(Cys759Phe) variant in homozygous state (5/14, 35.7%) referred hypoacusis at a relative old age (ranging from 50 to 76 years of age) and all of them reported mild hearing loss.

### Survival analysis

**Fig 1:**
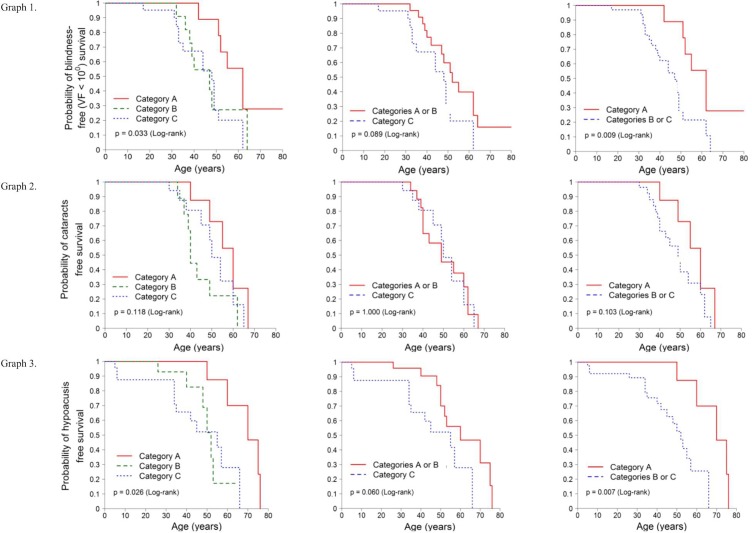
Survival analysis. Kaplan-Meier survival curves were estimated for each event and the curves of the different groups were compared using the log-rank test. The three categories of patients are considered separately, and then in two new regroupings (Category A + B) and (Category B + C). Category A: p.(Cys759Phe) homozygous, Category B: compound heterozygous p.(Cys759Phe) + *USH2A* missense variant, and Category C: compound heterozygous p.(Cys759Phe) + *USH2A* truncating variant. X axis: age in years. Y axis: probability of survival. Graph 1. Survival curve: fraction of patients free of legal blindness due to VF<10° over time. Graph 2. Survival curve: fraction of patients free of cataracts over time. Graph 3. Survival curve: fraction of patients free of hypoacusis over time.

The estimated survival curves for legal blindness (visual field, VF<10°), presence of cataracts and hypoacusis are shown in Fig 1.

In the case of legal blindness defined as VF<10°, the survival analysis revealed that there were differences between homozygous and heterozygous patients. Blindness takes less time to appear in heterozygous cases than in homozygous. Specifically, the median survival time is 62 years for homozygous compared to 49 years for heterozygous (Graph 1 in Fig 1).

There were no differences for cataracts (Graph 2 in Fig 1).

In the hypoacusis analysis, the difference also lied between homozygous and heterozygous patients, as it is shown in the Graph 3 (Fig 1). As for legal blindness due to VF loss, hearing loss takes less time to be perceived by compound heterozygous patients than by homozygous patients. Specifically, the median survival time was 70 years for homozygous and 53 years for heterozygous cases.

We found differences between genotype categories in the occurrence of legal blindness (VF) and hearing loss. By using the Cox proportional hazards models, we quantified how greater is the risk of presenting each of the two events in the heterozygous group compared to the homozygous patients. S3 Table shows the results of the Cox models for blindness (VF) and hearing loss. This analysis shows that the heterozygous group presents a statistically significant (three times, p = 0.014) higher risk of legal blindness (VF). In the case of hearing loss, the risk is >6 times higher for the heterozygous group (truncating + missense) than for the homozygous group (p = 0.017).

## Discussion

In this work, we have analyzed by different molecular approaches a large cohort of non-syndromic ARRP and USH2 patients. All the fifty-seven probands studied were characterized, 53 of them presenting the p.(Cys759Phe) variant both in homozygous or compound heterozygous state. Even though in the literature this variant has been reported in over 90 RP patients and 40 USH2 patients [6,11–14,21,25,27,41–47], Gonzalez-del Pozo *et al* have questioned the pathogenic role of this variant, at least when it appears homozygously [17]. Herein we report a total of 11 homozygous families (14 patients) for the p.(Cys759Phe) variant (S1 Fig). In these families, any other candidate variants in the *USH2A* gene or other RP genes that could explain the RP or USH2 phenotype were not found by our NGS analysis. Nevertheless, variants in deep-intronic or regulatory regions and complex rearrangements cannot be discarded.

The p.(Cys759Phe) variant has been identified in a heterozygous state in 262 cases, presenting a population frequency of 0.09% in the Genome Sequencing Project (GnomAD) and, although this variant has been reported with a higher prevalence in Spanish population [48], there is no homozygous cases in control population even in Spanish population databases.

Furthermore, based on the *in silico* prediction, several arguments point to destabilization of Cys759Phe mutated USH2A protein in the extracellular matrix by: i) the change of the polar Cys by the hydrophobic Phe in position 759, ii) the disruption of a disulfide bond and, iii) a certain misfolding in a region with a role in promoting interactions. If USH2A interactome is altered, the functions affected are mainly related to the ear and eye morphogenesis, visual perception (phototransduction) or retina homeostasis, among others (S1 Table).

All these evidences suggest that this variant may play a specific role in the pathogenesis of non-syndromic RP or USH2. However, further functional and gene editing studies [49] could shed additional light on the role of this *USH2A* variant.

This is the first work analyzing phenotypic differences in patients presenting the p.(Cys759Phe) variant. Clinical differences between genotypes are not easy to assess. Nevertheless, large cohorts and systematic recording of phenotypic data across time are required to confirm a defined genotype-phenotype correlation, as we underline here [50–53]. In our analysis, it has been observed that visual impairment in p.(Cys759Phe) homozygous patients appears to be milder than in heterozygous patients, and that truncating variants seem to lead to a more severe visual alteration, with legal blindness due to VF loss occurring more than 10 years later in homozygous.

Hearing loss in patients with a truncating variant is also more severe when compared with both p.(Cys759Phe) homozygous patients and missense heterozygous patients, as already reported by Blanco-Kelly *et al*. [15].

Moreover, relative early onset (early 40´s to early 50´s) of cataracts (which is a relative frequent feature of RP) [54,55], with no differences between the three groups, but later than that reported for non-p.(Cys759Phe) *USH2A* patients (30´s) [15], again supports that p.(Cys759Phe) is responsible for a milder phenotype than other *USH2A* variants.

Given the statistical differences between homozygous and non-homozygous cases, we can say that the onset of visual symptoms and diagnosis of audiological impairment occur later in homozygous patients. High variability in sensorineural hearing loss for p.(Cys759Phe) has already been described [7,23]; however, hearing loss in homozygous patients is relatively infrequent (5/14). Regarding the patient-referred age at onset (7th decade of life) we cannot exclude that these patients suffered from age related hearing loss, rather than a hearing impairment due to *USH2A* defect.

Additionally, we observe that the p.(Cys759Phe) carrier patients with RP due to another RP gene have a visual phenotype characterized by a far earlier onset than the exhibited by our p.(Cys759Phe) cohort. None of them presented hypoacusis, and visual phenotype is coincident with that reported for their causative genes [22,33–35], thus suggesting that the p.(Cys759Phe) is not contributing to their RP. Besides, we believe that it is important to highlight that when coming across patients with the p.(Cys759Phe) variant and an early-adulthood onset of a RD phenotype, further genetic analysis of non-*USH2A* RP genes is recommended.

In conclusion, the p.(Cys759Phe) variant must be considered as pathogenic, since this variant, in coexistence with other pathogenic *USH2A* alleles, rendered in all cases a symptomatic phenotype, even though p.(Cys759Phe) variant might be related to a less severe ocular disease course than patients with other *USH2A* mutations. Moreover, the comprehensive molecular analysis of our homozygous and compound heterozygous p.(Cys759Phe) patients, did not reveal other candidate RP genes as responsible for their phenotype in most of the families analyzed by NGS means. Only in four out of 57 families with only one p.(Cys759Phe) allele, a different RP gene (not *USH2A*) was responsible for the disease, indicating that they were simply carriers of p.(Cys759Phe). Additionally, further findings only occurred in three of the NGS re-analyzed cases:

In family RP-0605, re-analysis with NGS uncovered the presence of two coexisting retinal diseases (RP and cone affectation), since biallelic pathogenic variants in two different RD-related genes (*USH2A* and *CNGB3*) have been identified. These facts bring into consideration the importance of, once the genotype is known, to go back to the phenotype, or curating the phenotype when performing, analysing and reporting molecular studies, and when considering the enrolment of patients into clinical trials [56,57]; moreover, when genetic diseases co-existence has been reported to be present up to 4.9% of cases with informative whole-exome sequencing [58].

Additionally, family RP-0810 carried three *USH2A* pathogenic changes. Unfortunately we cannot predict whether the presence of two *in cis* missense variants is having a more severe impact in USH2A function than their impact alone.

NGS re-analysis of RP-0004 family has enabled us to detect one likely pathogenic allele in *PCDH15*. Nevertheless, we have not found a second allele in this gene. We would like to remark that our NGS analysis allows to us the study of CNV. Furthermore, mutations in *PCDH15* are related to Usher syndrome type I and to non-syndromic hearing loss [59]; phenotypes that are not keeping with our patient clinical findings.

As regards genotype-phenotype correlation associated with *USH2A* p.(Cys759Phe) variant, the presence of a p.(Cys759Phe) allele in homozygous state or in combination with other *USH2A* missense mutation is associated with a RP or a RP with a late onset of hypoacusis clinical subtypes. This is in line with that reported by Lenassi et al. [13]. In their study they found that some missense *USH2A* alleles (among them, the p.(Cys759Phe) variant) were confined to nonsyndromic RP cases, being enriched in nonsyndromic RP compared to USH2 cases, whereas "null" variants were rare in nonsyndromic cases and common in USH2 (as in the present series). However, they proposed a model of allele hierarchy of variants affecting USH2A function that does not fit with the results obtained in our study. In the model proposed by Lenassi, retinal-specific alleles would yield a non-syndromic RP phenotype when they appear in homozygous state or in combination with other retinal-specific or USH2-specific alleles. In the present study, all patients diagnosed as USH2 carried a null *USH2A* variant in compound heterozygous state with p.(Cys759Phe), being the former variant allegedly confined to retinal disease.

Additionally, there is a phenotype associated to p.(Cys759Phe) homozygosity consisting on a later diagnosis of RP and slower progression of VF loss, with a very late hypoacusis diagnosis (around 7^th^ decade).

In summary, our study objectively validates the pathogenicity of *USH2A* p.(Cys759Phe) and presents the clinical differences between p.(Cys759Phe) patients.

## Materials and methods

### Patients

Fifty-seven unrelated Spanish families diagnosed with RP or USH2 were recruited from the Biobank of the Fundación Jiménez Díaz Hospital (Madrid, Spain).

DNA was extracted from peripheral blood samples of index patients and their family members as described by Perez-Carro *et al* [22]. Informed consent was obtained from all subjects involved in this study. All procedures were reviewed and approved by the Ethics Committee of the hospital and adhered to the tenets of the Declaration of Helsinki and further reviews.

A total of 59 patients (from 52 families), presenting the p.(Cys759Phe) variant in homozygous (14/59) or in compound heterozygous (45/59) state were included in the phenotype analysis. Additionally, the phenotype of 4 patients (from 4 families) presenting the p.(Cys759Phe) variant in the *USH2A* gene and with pathogenic variants in the *RP1*(2), *PROM1* or *CNGB1* genes, are shown in S2 Table and they have not been statistically analyzed. Number of patients that underwent genetic and/or phenotype analysis are shown in S2 Fig.

### Genetic analysis

All probands were previously screened for known mutations with classical molecular techniques: a specific ARRP/Usher genotyping microarray (AsperBiotech, Tartu, Estonia; http://www.asperbio.com/asper-ophthalmics) or Sanger sequencing. For those cases with the p.(Cys759Phe) variant heterozygously, Sanger sequencing to screen the deep intronic c.7595-2144A>G variant in *USH2A* and multiplex ligation-dependent probe amplification (MLPA probemixes P361 and P362, MRC-Holland), in order to find large deletions/duplications, were performed following the manufacturer’s instructions and analyzing the results with the Coffalyser software (MRC-Holland, Amsterdam, The Netherlands).

In order to characterize the incompletely characterized patients and to exclude the implication of additional variants in other RD and hypoacusis genes (rather than those identified in *USH2A*), homozygous and heterozygous p.(Cys759Phe) patients were analyzed with different NGS approaches based on: a) Targeted-NGS, different in-house gene panels were used along this research, containing genes previously associated with IRD (RetNet); and b) Clinical exome -TruSightOne (Illumina) or Clinical Exome Solution (Sophia Genetics)- containing more than 4.500 genes associated with known clinical phenotypes (OMIM Database). Genes included in each panel and those selected for the analysis of the clinical exome are detailed in S4 Table.

Sanger sequencing, as reported elsewhere [22], was performed to confirm pathogenic variants and to segregate them in the families.

### Assessment of pathogenicity

Novel rare variants were checked in the 1000 Genomes Project, Exome Variant Server (EVS, version 0.0.30), Exome Aggregation Consortium (ExAC, version 0.3.1) and Genome Aggregation Database (gnomAD, version r2.0.2). Furthermore, 267 in-house whole exome from Spanish healthy individuals (CIBERER Collaborative Spanish Variant Server) were used to evaluate the frequency of the variants found in this study.

Four different predictive software programs were used to assess the pathogenesis of the missense variants: 1) Sorting Intolerant from Tolerant (SIFT), 2) Polymorphism Phenotyping v2 (Polyphen-2), 3) Align GVGD and 4) Mutation Taster. Those variants predicted as damaging by at least two different out of four prediction softwares were considered as a possible causative variant.

To check the effect of p.(Cys759Phe) in the USH2A structure and function, USMA (https://neuro-2.iurc.montp.inserm.fr/USMA/) and Uniprot, and PTMcode v.2 [38] databases were used, respectively.

For the prediction of the functional impact on the USH2A neighbourhood, we used STRING facility [39].

### Clinical examination

All patients were classified according to their clinical history, pedigree data, results from ophthalmological studies [60–63], audiological test results (or self-reported hearing loss) and neurophysiological and vestibular test results [63–65].

A defined protocol, previously described by Blanco-Kelly *et al*. [15], was followed to collect the data for establishing the ophthalmological status.

The severity of visual impairment was established both for VA loss and VF loss, and classified following the WHO (World Health Organization) criteria, as detailed by Blanco-Kelly *et al*. [66].

Hearing loss severity was categorized according to audiological tests [63–65] as reported by Blanco-Kelly *et al*. [15].

Patients with mutations only in *USH2A* were classified in one of the three following groups: "ARRP", defined as RP and absence of hypoacusis (based on last audiogram or at clinical interrogation) at the time of assessment; "USH2", defined as RP plus hypoacusis with an USH2 audiogram and/or self-reported hypoacusis at early age of onset; and "RP+hypoacusis", defined as RP plus hypoacusis with non-USH2 audiogram and/or late age of onset. An USH2 audiogram is defined when it showed a neurosensorial and bilateral hearing loss, with mild-to-moderate loss at low and middle frequencies and moderate-to-profound loss at high frequencies. As an example, audiometries of two USH2 patients included in this study (RP-0061 and RP-1031) are depicted in S3 Fig.

### Statistical analysis

The statistical analysis was performed for the patients presenting *USH2A* as responsible for their condition. The differences between values for the analyzed phenotypic aspects were tested by the Student´s t test. The differences in the frequency of the genotypes within the 3 types of phenotypes (ARRP/SRP, USH2 and RP+hypoacusis) were analysed by calculating odds ratio and χ^2^ or two-tailed Fisher’s test; when non-applicable, Sheskin D.J. (2004) [40] was applied.

Survival analysis for VF, cataracts and hypoacusis were estimated by the Kaplan-Meier method for each event, and the curves of the different groups were compared using the log-rank test. The three categories of patients were considered separately, and then in two new regroupings (Category A + B) and (Category B + C). Risk differences were estimated using Cox proportional hazards regression models.

## Supporting information

S1 Fig Pedigree of homozygous and heterozygous families for *USH2A* p.(Cys759Phe) variant. (A) Pedigrees of homozygous p.(Cys759Phe) families. (B) Pedigrees of compound heterozygous families. (C) Pedigrees of families with causative mutations in other RP genes. Co-segregation analyses in family members are displayed when available. Abbreviations: m1, p.(Cys759Phe) allele; m2, second mutated allele in *USH2A*; m, mutated allele in other non-*USH2A* RD gene; wt, wild-type allele; NA, DNA not available.
(DOC)

S2 Fig Number of patients/families that underwent different analysis during this study. *Three patients (RP-1574, RP-0391 and RP-1016/982) initially characterized using classical techniques were not re-analyzed by NGS due to lack of sample with enough quantity and/or quality. ** RP-2424 was excluded from phenotype studies, since no clinical information was available.
(DOC)

S3 Fig Audiograms from two Usher type II patients (families RP-0061 and RP-1031). Audiograms show a typical Usher type II down-sloping pattern with bilateral hypoacusis from moderate to severe degree at high frequencies.
(DOC)

S1 Table Gene Ontology terms (biological process) enriched in USH2A interactome. USH2A interacting proteins (with a STRING combined score > 0.400) compared to the whole genome. Abbreviations: GO, gene ontology; FDR, false discovery rate.
(DOC)

S2 Table Clinical features of 63 patients from 56 families with at least one p.(Cys759Phe) allele. Proband from family RP-2424 was excluded from this table, since only molecular information was available. Families are organized in categories: category A, homozygous patients for p.(Cys759Phe) allele; category B, compound heterozygous patients p.(Cys759Phe) + *USH2A* missense mutation; category C, compound heterozygous patients p.(Cys759Phe) + *USH2A* truncating mutation; and families with pathogenic variants in other RP genes.
Abbreviations: Hom, homozygous; CH, compound heterozygous; Het, heterozygous; ARRP, autosomal recessive retinitis pigmentosa; SRP, sporadic retinitis pigmentosa; NB: night blindness; VF: visual field; VA: visual acuity; BCVA: best corrected visual acuity; CF: counting finger; LP: light perception; NLP: no light perception; NA: not availaible; y: years; IOP: intraocular pressure; EOG: electrooculagram; ERG: electroretinogram; MA: macular alteration; NR: non-recordable; RA: reduced amplitude; DL: delayed latencies; Sco: scotopic; Pho: photopic; OCT: optical coherence tomography; AR: Arden ratio; FA: fluorescent angiography; Typical RP fundus: pale optic disc, narrowed retina vessels and pigmentary changes (bone-spicules); OD: right eye; OS: left eye; BE: both eyes; RPE: Retinal Pigment Epithelium; —> progression; * Hypoacusis (age at last examination): audiogram and/or self-reported hypoacusis under clinical interrogation; RE: right ear; LE: left ear; Infancy^#^ (≤6y): referred "infancy" was considered less than or equal to 6 years (for statistical analysis, infancy = 6 years); 1: mild hearing loss; 2: moderate hearing loss; 3: severe/profound hearing loss.
(DOC)

S3 Table Cox proportional hazards models. The table shows the results of the Cox models for the risk of presenting blindness and hearing loss, comparing p. (Cys759Phe) heterozygous patients (missense + truncating) with p. (Cys759Phe) homozygous patients. The models are summarized by the hazard ratio, its 95% confidence interval and the p value.
Abbreviations: VF, visual field; HR, hazard ratio; CI, confidence interval.
(DOC)

S4 Table Genes related to IRD included in the different NGS approaches used during this study. (A) In house IRD_NGS panel with 68 genes. (B) In house IRD_NGS panel with 75 genes. (C) Virtual panel with 205 genes selected for clinical exome analysis. (D) Virtual panel with 83 genes associated with deafness.
(DOC)
