# Supplementary material for: Unravelling the pathogenic role and genotype-phenotype correlation of the USH2A p.(Cys759Phe) variant among Spanish families
Source: PLoS One. 2018 Jun 18;13(6):e0199048. doi: 10.1371/journal.pone.0199048 (PMC6005481; doi:10.1371/journal.pone.0199048)
Supplement: S1 Fig — (A) Pedigrees of homozygous p.(Cys759Phe) families. (B) Pedigrees of compound heterozygous families. (C) Pedigrees of families with causative mutations in other RP genes. Co-segregation analyses in family members are displayed when available. Abbreviations: m1, p.(Cys759Phe) allele; m2, second mutated allele in USH2A; m, mutated allele in other non-USH2A RD gene; wt, wild-type allele; NA, DNA not available. (DOC) [file pone.0199048.s001.doc]

**SUPPORTING INFORMATION**

**S1 Fig. Pedigree of homozygous and heterozygous families for *USH2A* p.(Cys759Phe) variant.** (A) Pedigrees of homozygous p.(Cys759Phe) families. (B) Pedigrees of compound heterozygous families. (C) Pedigrees of families with causative mutations in other RP genes. Co-segregation analyses in family members are displayed when available. Abbreviations: m1, p.(Cys759Phe) allele; m2, second mutated allele in *USH2A*; m, mutated allele in other non-*USH2A* RP gene; wt, wild-type allele; NA, DNA not available.

S1 Fig:

A. HOMOZYGOUS FAMILIES:

B. COMPOUND HETEROZYGOUS FAMILIES:

C. FAMILIES WITH CAUSATIVE MUTATIONS IN OTHER RP GENES:
