# Supplementary material for: Unravelling the pathogenic role and genotype-phenotype correlation of the USH2A p.(Cys759Phe) variant among Spanish families
Source: PLoS One. 2018 Jun 18;13(6):e0199048. doi: 10.1371/journal.pone.0199048 (PMC6005481; doi:10.1371/journal.pone.0199048)
Supplement: S2 Fig — *Three patients (RP-1574, RP-0391 and RP-1016/982) initially characterized using classical techniques were not re-analyzed by NGS due to lack of sample with enough quantity and/or quality. ** RP-2424 was excluded from phenotype studies, since no clinical information was available. (DOC) [file pone.0199048.s002.doc]

**SUPPORTING INFORMATION**

**S2 Fig. Number of patients/families that underwent different analysis during this study.** *Three patients (RP-1574, RP-0391 and RP-1016/982) initially characterized using classical techniques were not re-analyzed by NGS due to lack of sample with enough quantity and/or quality. ** RP-2424 was excluded from phenotype studies since no clinical information was available.

S2 Fig:
