# Supplementary material for: Unravelling the pathogenic role and genotype-phenotype correlation of the USH2A p.(Cys759Phe) variant among Spanish families
Source: PLoS One. 2018 Jun 18;13(6):e0199048. doi: 10.1371/journal.pone.0199048 (PMC6005481; doi:10.1371/journal.pone.0199048)

**SUPPORTING INFORMATION**

**S3 Fig. Audiograms from two Usher type II patients (families RP-0061 and RP-1031).** Audiograms show a typical Usher type II down-sloping pattern with bilateral hypoacusis from moderate to severe degree at high frequencies.

S3 Fig:


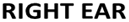

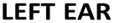

Supplement: S3 Fig — Audiograms show a typical Usher type II down-sloping pattern with bilateral hypoacusis from moderate to severe degree at high frequencies. (DOC) [file pone.0199048.s003.doc]
