# Supplementary material for: Unravelling the pathogenic role and genotype-phenotype correlation of the USH2A p.(Cys759Phe) variant among Spanish families
Source: PLoS One. 2018 Jun 18;13(6):e0199048. doi: 10.1371/journal.pone.0199048 (PMC6005481; doi:10.1371/journal.pone.0199048)
Supplement: S1 Table — USH2A interacting proteins (with a STRING combined score > 0.400) compared to the whole genome. Abbreviations: GO, gene ontology; FDR, false discovery rate. (DOC) [file pone.0199048.s004.doc]

**SUPPORTING INFORMATION**

**S1 Table. Gene Ontology terms (biological process) enriched in USH2A interactome.** USH2A interacting proteins (with a STRING combined score > 0.400) compared to the whole genome. Abbreviations: GO, gene ontology; FDR, false discovery rate.

**S1 Table. Gene Ontology terms (biological process) enriched in USH2A interactome.**

| **GO id** | **GO name** | **Number of genes from**  **USH2A interactome annotated** | **FDR** |
| --- | --- | --- | --- |
| GO:0007605 | sensory perception of sound | 14 | 2.16E-15 |
| GO:0045494 | photoreceptor cell maintenance | 10 | 4.44E-15 |
| GO:0001895 | retina homeostasis | 11 | 1.91E-14 |
| GO:0050953 | sensory perception of light stimulus | 13 | 2.99E-12 |
| GO:0050957 | equilibrioception | 6 | 2.99E-12 |
| GO:0007600 | sensory perception | 20 | 6.44E-12 |
| GO:0050877 | neurological system process | 21 | 6.30E-11 |
| GO:0048839 | inner ear development | 10 | 1.61E-08 |
| GO:0003008 | system process | 21 | 2.08E-08 |
| GO:0048871 | multicellular organismal homeostasis | 11 | 2.54E-08 |
| GO:0043583 | ear development | 10 | 3.57E-08 |
| GO:0060113 | inner ear receptor cell differentiation | 7 | 3.81E-08 |
| GO:0060119 | inner ear receptor cell development | 6 | 2.34E-07 |
| GO:0007601 | visual perception | 9 | 7.74E-07 |
| GO:0060122 | inner ear receptor stereocilium organization | 5 | 0.00000132 |
| GO:0050885 | neuromuscular process controlling balance | 6 | 0.00000285 |
| GO:0035315 | hair cell differentiation | 5 | 0.0000166 |
| GO:0001754 | eye photoreceptor cell differentiation | 5 | 0.0000312 |
| GO:0090596 | sensory organ morphogenesis | 8 | 0.0000572 |
| GO:0042592 | homeostatic process | 15 | 0.0000587 |
| GO:0042472 | inner ear morphogenesis | 6 | 0.0000651 |
| GO:0042471 | ear morphogenesis | 6 | 0.00016 |
| GO:0009887 | organ morphogenesis | 12 | 0.000195 |
| GO:0042462 | eye photoreceptor cell development | 4 | 0.00033 |
| GO:0042491 | auditory receptor cell differentiation | 4 | 0.000421 |
| GO:0048666 | neuron development | 11 | 0.000603 |
| GO:0007423 | sensory organ development | 9 | 0.000753 |
| GO:0009581 | detection of external stimulus | 6 | 0.00144 |
| GO:0009582 | detection of abiotic stimulus | 6 | 0.00155 |
| GO:0009584 | detection of visible light | 5 | 0.0017 |
| GO:0048562 | embryonic organ morphogenesis | 7 | 0.0017 |
| GO:0048598 | embryonic morphogenesis | 9 | 0.0017 |
| GO:0030182 | neuron differentiation | 11 | 0.00299 |
| GO:0060117 | auditory receptor cell development | 3 | 0.00299 |
| GO:0030030 | cell projection organization | 11 | 0.00309 |
| GO:0048592 | eye morphogenesis | 5 | 0.00594 |
| GO:0000086 | G2/M transition of mitotic cell cycle | 5 | 0.00664 |
| GO:0065008 | regulation of biological quality | 18 | 0.0107 |
| GO:0009790 | embryo development | 10 | 0.0126 |
| GO:0048568 | embryonic organ development | 7 | 0.0176 |
| GO:0007399 | nervous system development | 14 | 0.0196 |
| GO:0007602 | phototransduction | 4 | 0.0307 |
| GO:0009416 | response to light stimulus | 6 | 0.0307 |
| GO:0045184 | establishment of protein localization | 11 | 0.0307 |
| GO:0048513 | organ development | 16 | 0.0315 |
| GO:0048699 | generation of neurons | 11 | 0.033 |
| GO:0009628 | response to abiotic stimulus | 10 | 0.037 |
| GO:0048468 | cell development | 12 | 0.039 |

USH2A interacting proteins (with a STRING combined score > 0.400) compared to the whole genome.

Abbreviations: GO, gene ontology; FDR, false discovery rate.
