# Supplementary material for: Unravelling the pathogenic role and genotype-phenotype correlation of the USH2A p.(Cys759Phe) variant among Spanish families
Source: PLoS One. 2018 Jun 18;13(6):e0199048. doi: 10.1371/journal.pone.0199048 (PMC6005481; doi:10.1371/journal.pone.0199048)
Supplement: S2 Table — Proband from family RP-2424 was excluded from this table, since only molecular information was available. Families are organized in categories: category A, homozygous patients for p.(Cys759Phe) allele; category B, compound heterozygous patients p.(Cys759Phe) + USH2A missense mutation; category C, compound heterozygous patients p.(Cys759Phe) + USH2A truncating mutation; and families with pathogenic variants in other RP genes. Abbreviations: Hom, homozygous; CH, compound heterozygous; Het, heterozygous; ARRP, autosomal recessive retinitis pigmentosa; SRP, sporadic retinitis pigmentosa; NB: night blindness; VF: visual field; VA: visual acuity; BCVA: best corrected visual acuity; CF: counting finger; LP: light perception; NLP: no light perception; NA: not availaible; y: years; IOP: intraocular pressure; EOG: electrooculagram; ERG: electroretinogram; MA: macular alteration; NR: non-recordable; RA: reduced amplitude; DL: delayed latencies; Sco: scotopic; Pho: photopic; OCT: optical coherence tomography; AR: Arden ratio; FA: fluorescent angiography; Typical RP fundus: pale optic disc, narrowed retina vessels and pigmentary changes (bone-spicules); OD: right eye; OS: left eye; BE: both eyes; RPE: Retinal Pigment Epithelium; —> progression; * Hypoacusis (age at last examination): audiogram and/or self-reported hypoacusis under clinical interrogation; RE: right ear; LE: left ear; Infancy# (≤6y): referred "infancy" was considered less than or equal to 6 years (for statistical analysis, infancy = 6 years); 1: mild hearing loss; 2: moderate hearing loss; 3: severe/profound hearing loss. (DOC) [file pone.0199048.s005.doc]

**SUPPORTING INFORMATION**

**S2 Table. Clinical features of 63 patients from 56 families with at least one p.(Cys759Phe) allele.** Proband from family RP-2424 was excluded from this table, since only molecular information was available. Families are organized in categories: category A, homozygous patients for p.(Cys759Phe) allele; category B, compound heterozygous patients p.(Cys759Phe) + USH2A missense mutation; category C, compound heterozygous patients p.(Cys759Phe) + USH2A truncating mutation; and families with pathogenic variants in other RP genes.

Abbreviations: Hom, homozygous; CH, compound heterozygous; Het, heterozygous; ARRP, autosomal recessive retinitis pigmentosa; SRP, sporadic retinitis pigmentosa; NB: night blindness; VF: visual field; VA: visual acuity; BCVA: best corrected visual acuity; CF: counting finger; LP: light perception; NLP: no light perception; NA: not availaible; y: years; IOP: intraocular pressure; EOG: electrooculagram; ERG: electroretinogram; MA: macular alteration; NR: non-recordable; RA: reduced amplitude; DL: delayed latencies; Sco: scotopic; Pho: photopic; OCT: optical coherence tomography; AR: Arden ratio; FA: fluorescent angiography ; Typical RP fundus: pale optic disc, narrowed retina vessels and pigmentary changes (bone-spicules); OD: right eye; OS: left eye; BE: both eyes; RPE: Retinal Pigment Epithelium; --> progression; * Hypoacusis (age at last examination): audiogram and/or self-reported hypoacusis under clinical interrogation; RE: right ear; LE: left ear; Infancy# (≤6y): referred "infancy" was considered less than or equal to 6 years (for statistical analysis, infancy=6 years); 1: mild hearing loss; 2: moderate hearing loss; 3: severe/profound hearing loss.

**S2 Table. Clinical features of 63 patients from 56 families with at least one p.(Cys759Phe) allele.** Families from category A.

**S2 Table (continued).** Families from category B.

**S2 Table (continued).** Families from category C.

**S2 Table (continued).** Families from category C.

**S2 Table (continued).** Families with mutations in other RP genes.

Proband from family RP-2424 was excluded from this table, since only molecular information was available. Families are organized in categories: category A, homozygous patients for p.(Cys759Phe) allele; category B, compound heterozygous patients p.(Cys759Phe) + *USH2A* missense mutation; category C, compound heterozygous patients p.(Cys759Phe) + *USH2A* truncating mutation; and families with pathogenic variants in other RP genes.

Abbreviations: Hom, homozygous; CH, compound heterozygous; Het, heterozygous; ARRP, autosomal recessive retinitis pigmentosa; SRP, sporadic retinitis pigmentosa; NB: night blindness; VF: visual field; VA: visual acuity; BCVA: best corrected visual acuity; CF: counting finger; LP: light perception; NLP: no light perception; NA: not available; y: years; IOP: intraocular pressure; EOG: electrooculogram; ERG: electroretinogram; MA: macular alteration; NR: non-recordable; RA: reduced amplitude; DL: delayed latencies; Sco: scotopic; Pho: photopic; OCT: optical coherence tomography; AR: Arden ratio; FA: fluorescent angiography ; Typical RP fundus: pale optic disc, narrowed retina vessels and pigmentary changes (bone-spicules); OD: right eye; OS: left eye; BE: both eyes; RPE: Retinal Pigment Epithelium; --> progression; * Hypoacusis (age at last examination): audiogram and/or self-reported hypoacusis under clinical interrogation; RE: right ear; LE: left ear; Infancy# (≤6y): referred "infancy" was considered less than or equal to 6 years (for statistical analysis, infancy=6 years); 1: mild hearing loss; 2: moderate hearing loss; 3: severe/profound hearing loss.
