# Supplementary material for: Unravelling the pathogenic role and genotype-phenotype correlation of the USH2A p.(Cys759Phe) variant among Spanish families
Source: PLoS One. 2018 Jun 18;13(6):e0199048. doi: 10.1371/journal.pone.0199048 (PMC6005481; doi:10.1371/journal.pone.0199048)
Supplement: S3 Table — The table shows the results of the Cox models for the risk of presenting blindness and hearing loss, comparing p. (Cys759Phe) heterozygous patients (missense + truncating) with p. (Cys759Phe) homozygous patients. The models are summarized by the hazard ratio, its 95% confidence interval and the p value. Abbreviations: VF, visual field; HR, hazard ratio; CI, confidence interval. (DOC) [file pone.0199048.s006.doc]

**SUPPORTING INFORMATION**

**S3 Table. Cox proportional hazards models.** The table shows the results of the Cox models for the risk of presenting blindness and hearing loss, comparing p. (Cys759Phe) heterozygous (missense + truncating) with p. (Cys759Phe) homozygous. The models are summarized by the hazard ratio, its 95% confidence interval and the p value.

**S3 Table. Cox proportional hazards models.**

| **Event** | **HR** | **(95% CI)** | **P** |
| --- | --- | --- | --- |
| VF <10^o^ | 3.21 | (1.27, 8.14) | 0.014 |
| Hypoacusis | 6.22 | (1.39, 27.9) | 0.017 |

The table shows the results of the Cox models for the risk of presenting blindness and hearing loss, comparing p.(Cys759Phe) heterozygous (missense + truncating) patients with p.(Cys759Phe) homozygous patients. The models are summarized by the hazard ratio, its 95% confidence interval and the p value.

Abbreviations: VF, visual field; HR, hazard ratio; CI, confidence interval.
