# Supplementary material for: Unravelling the pathogenic role and genotype-phenotype correlation of the USH2A p.(Cys759Phe) variant among Spanish families
Source: PLoS One. 2018 Jun 18;13(6):e0199048. doi: 10.1371/journal.pone.0199048 (PMC6005481; doi:10.1371/journal.pone.0199048)
Supplement: S4 Table — (A) In house IRD_NGS panel with 68 genes. (B) In house IRD_NGS panel with 75 genes. (C) Virtual panel with 205 genes selected for clinical exome analysis. (D) Virtual panel with 83 genes associated with deafness. (DOC) [file pone.0199048.s007.doc]

**SUPPORTING INFORMATION**

**S4 Table.** **Genes related to RD included in the different NGS approaches used during this study.** (A) In house RD_NGS panel with 68 genes. (B) In house RD_NGS panel with 75 genes. (C) Virtual panel with 205 genes selected for clinical exome analysis. (D) Virtual panel with 83 genes associated with deafness.

**S4 Table. Genes related to IRD included in the different NGS approaches used during this study.**

A. In house IRD_NGS panel with 68 genes

B. In house IRD_NGS panel with 75 genes

C. Virtual panel with 205 genes selected for clinical exome analysis

D. Virtual panel with 83 genes associated with deafness.
